# Identification and sequence determination of a new chrysovirus infecting the phytopathogenic fungus *Dothistroma septosporum*

**DOI:** 10.1007/s00705-023-05768-9

**Published:** 2023-04-18

**Authors:** Unnati A. Shah, John O. Daudu, Charalampos Filippou, Katherine V. Tubby, Robert H. A. Coutts, Ioly Kotta-Loizou

**Affiliations:** 1 Department of Clinical, Pharmaceutical and Biological Science, School of Life and Medical Sciences, University of Hertfordshire, Hatfield, AL10 9AB UK; 2 Forest Research, Alice Holt Lodge, Wrecclesham, Farnham, GU10 4LH UK; 3 Department of Life Sciences, Faculty of Natural Sciences, Imperial College London, Imperial College Road, London, SW7 2AZ UK

## Abstract

A new double-stranded (ds) RNA mycovirus has been identified in isolate Ds752-1 of the phytopathogenic fungus *Dothistroma septosporum*, the causal agent of Dothistroma needle blight, also known as red band needle blight or pine needle blight. Dothistroma septosporum chrysovirus 1 (DsCV-1) is a new member of the genus *Alphachrysovirus* in the family *Chrysoviridae*. The DsCV-1 genome comprises four dsRNA elements designated 1, 2, 3, and 4 from largest to smallest. dsRNA1 encodes an RNA-dependent RNA polymerase (RdRP) that is most similar to the RdRP of Erysiphe necator associated chrysovirus 3. dsRNA2 potentially encodes two hypothetical proteins, one of which is small and has no homology to known proteins, and one of which is large with significant sequence similarity to the alphachryso-P3 of other alphachrysoviruses. dsRNA3 and dsRNA4 encode a coat protein (CP) and a putative cysteine protease, respectively. This is the first report of a mycovirus infecting the fungus *D. septosporum*, and DsCV-1 is one of three *Chrysoviridae* family members found to possess genomic dsRNAs potentially encoding more than one protein.

**Supplementary Information:**

The online version contains supplementary material available at 10.1007/s00705-023-05768-9.

Fungal viruses, or mycoviruses, are widespread across the major taxa of fungi [1]. The diversity of known mycoviruses has increased rapidly over the last few years, mainly due to the development and widespread use of state-of-the-art next-generation sequencing techniques. Currently, the International Committee on Taxonomy of Viruses officially recognizes over 25 taxa, 10 of which are families or genera accommodating mycoviruses with double-stranded (ds) RNA genomes (*Amalga-*, *Chryso*-, *Curvula-*, *Megabirna*-, *Partiti*-, *Polymyco-*, *Quadri*-, *Spinareo*-, and *Totiviridae* and *Botybirnavirus*). Additionally, there are still many taxonomically unclassified dsRNA mycoviruses [1, 2]. dsRNA mycoviruses do not have an extracellular phase in their replication cycle and are not vectored but instead rely on horizontal transmission through anastomosis and/or vertical transmission through spores [2]. Despite some of them being asymptomatic in their hosts, the development of mycoviruses as biological control agents is of great interest, since they may reduce [3] or increase [4] the virulence of their fungal hosts.

In Britain, Dothistroma needle blight, also known as red band needle blight or pine needle blight, is caused by the fungus *Dothistroma septosporum*. *D. septosporum* has been found on a range of conifer species, but pines are its most common hosts. Defoliation can continue annually and gradually weaken the tree, significantly reducing timber yields and resulting in tree death [5]. No mycovirus infections have been reported previously in *D. septosporum*. In this study, we report a new member of the genus *Alphachrysovirus* in the family *Chrysoviridae*, designated "Dothistroma septosporum chrysovirus 1" (DsCV-1).

*D. septosporum* isolate Ds752.1, which harbors DsCV-1, was isolated in 2012 in West Argyll, Scotland, from Corsican pine and was identified morphologically and by molecular analysis as mating type 1. dsRNA elements were extracted from fresh mycelium and treated with DNase I and S1 nuclease prior to separation by agarose gel electrophoresis, together with DNA size standards (HyperLadder I; Bioline), as indicated in Fig. 1a. Purified dsRNA was used as a template for random polymerase chain reaction (PCR) amplification, genome walking, and RNA-ligase-mediated rapid amplification of cDNA ends (RLM RACE) [6]. All products were cloned and sequenced as described previously [4].

**Fig. 1:**
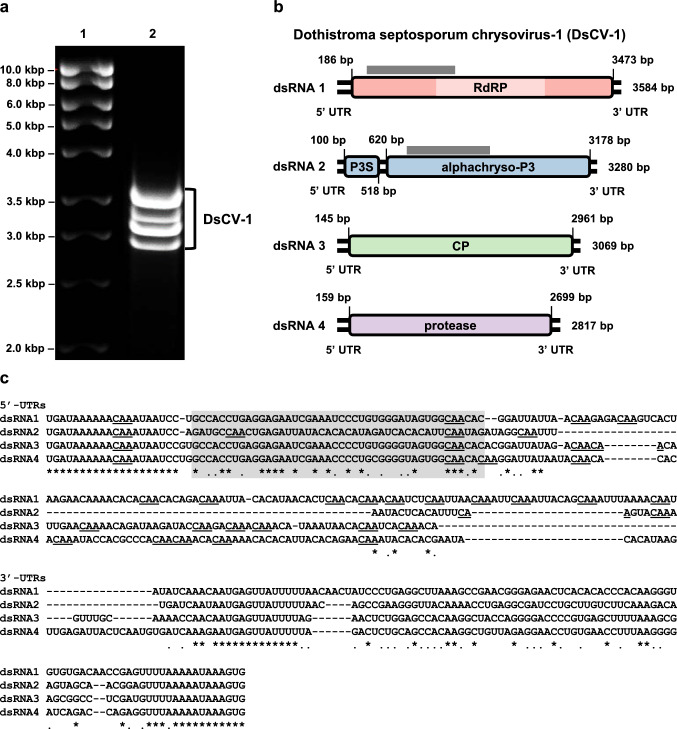
(a) Agarose gel electrophoresis of dsRNA elements present in *D. septosporum* isolate Ds752-1 (lane 2), ranging between 2.8 and 3.6 kbp based on HyperLadder I (lane 1). (b) Genome organization of DsCV-1. The genome consists of four dsRNA segments and open reading frames (ORFs) are represented by rectangular boxes. The light-coloured box in dsRNA 1 indicates the viral RdRP motifs (PF02123). The grey boxes above dsRNAs 1 and 2 indicate high sequence similarity between the RdRP and the alphachryso-P3. (c) Nucleotide sequence alignment of the DsCV-1 5’- and 3’-UTRs. The “box 1” region is highlighted in grey, and the CAA repeats in the 5’-UTRs are underlined. Asterisks and dots indicate conservation of all four and three out of four nucleotides, respectively.

The genome organization of DsCV-1 is shown in Fig. 1b, and the sequences have been deposited in public databases (project accession number PRJEB57200). The dsRNA elements comprising the genome were numbered 1 to 4 from largest to smallest. dsRNA1 is 3584 bp in length (GC content, 44.61%), contains a single open reading frame (ORF) encoding a 1095-amino-acid (aa) protein (126.45 kDa) with eight conserved motifs that are typically present in RNA-dependent RNA polymerases (RdRPs) of eukaryotic dsRNA viruses [7], and shows a high degree of sequence similarity to alphachrysovirus RdRPs. dsRNA2 is 3280 bp in length (GC content, 45.25%) and has two ORFs, which potentially encode 141- and 852-aa proteins, 15.80 kDa and 95.83 kDa in size, respectively. BLAST searches showed, for the latter, a high degree of sequence similarity to hypothetical alphachryso-P3 proteins of unknown function, while for the former, designated alphachryso-P3S (S for small), no similarity to any database-accessed protein sequences was found. As for other alphachrysoviruses, the hypothetical alphachryso-P3 protein contains a ‘‘phytoreovirus S7 domain’’, found in viral core proteins with nucleic acid binding activity [2, 8], and its N-terminal region shares a degree of sequence similarity with comparable N-terminal regions of alphachrysovirus RdRPs. dsRNA3 is 3069 bp in length (GC content, 46.97%), containing an ORF that encodes a 938-aa protein (104.21 kDa) with a high degree of sequence similarity to alphachrysovirus capsid protein (CP) genes. dsRNA4 is 2817 bp in length (GC content, 47.80%) and contains an ORF that encodes an 846-aa protein (93.49 kDa) with motifs typical of cysteine proteases and a high degree of sequence similarity to putative proteases of alphachrysoviruses.

BLAST searches showed that all DsCV-1 proteins had the highest sequence similarity to those of Erysiphe necator associated chrysovirus 3 (EnACV-3): 71%, 44%, 51%, and 62% for the RdRP, alphachryso-P3, CP, and protease, respectively. The nucleotide sequences of the alphachryso-P3-encoding segments from DsCV-1, EnACV-3, and some other chrysoviruses such as Cryphonectria nitschkei chrysovirus 1 (CnCV-1) [8] are longer than the respective CP-encoding segments, but the significance of this feature is unknown. Interestingly, ORFs putatively encoding proteins between 110 and 250 aa in length, similar in size and location to alphachryso-P3S, could be predicted from the sequence of alphachryso-P3-encoding dsRNA from EnACV-3 and CnCV-1. However, the sequences of the putative proteins are not related, and the position of the upstream ORF is different with respect to the alphachryso-P3 ORF: -1/+2 for DsCV-1, -2/+1 for EnACV-3, and in frame for CnCV-1.

The termini of the 5’ and 3’ untranslated regions (UTRs) flanking the ORFs are highly conserved among the four DsCV-1 dsRNAs (Fig. 1c). The 5’-UTRs of dsRNAs 1-4 are 189, 99, 144, and 158 nt in length, respectively. They exhibit a highly conserved region of *ca.* 45 nt, similar to ‘‘box 1’’ found in most chrysoviruses [9], and a second region containing numerous CAA repeats immediately downstream from ‘‘box 1’’, similar to the enhancer elements found in the 5’-UTRs of tobamoviruses [9, 10]. The 3’-UTRs of dsRNAs 1-4 are 109, 107, 102, and 117 nt in length, respectively (Fig. 1c).

Phylogenetic analysis based on the complete or almost complete aa sequence of the RdRP genes of DsCV-1 and selected members of the family *Chrysoviridae* was performed using the programme MEGA 11 [11]. A multiple alignment of RdRP aa sequences was produced using MUSCLE as implemented in MEGA 11, all positions with less than 30% site coverage were eliminated, and the LG+G+I+F substitution model was used. A maximum-likelihood (ML) phylogenetic tree was constructed in MEGA 11 with 100 bootstrap replicates. The ML phylogenetic tree revealed that the putative RdRP encoded by DsCV-1 most strongly resembled those of viruses within the genus *Alphachrysovirus*, family *Chrysoviridae* (Fig. 2). Based on the established chrysovirus species demarcation criteria, including the novel host *D. septosporum*, the unusually long 5’-UTR of dsRNA2, and the <70% aa sequence identity in the RdRP to representatives of already recognised species, DsCV-1 should be considered a member of a new species, for which we propose the name "*Alphachrysovirus dothistromae*". The potential pathogenic effects of DsCV-1 in *D. septosporum* were not evaluated in the present work.

**Fig. 2:**
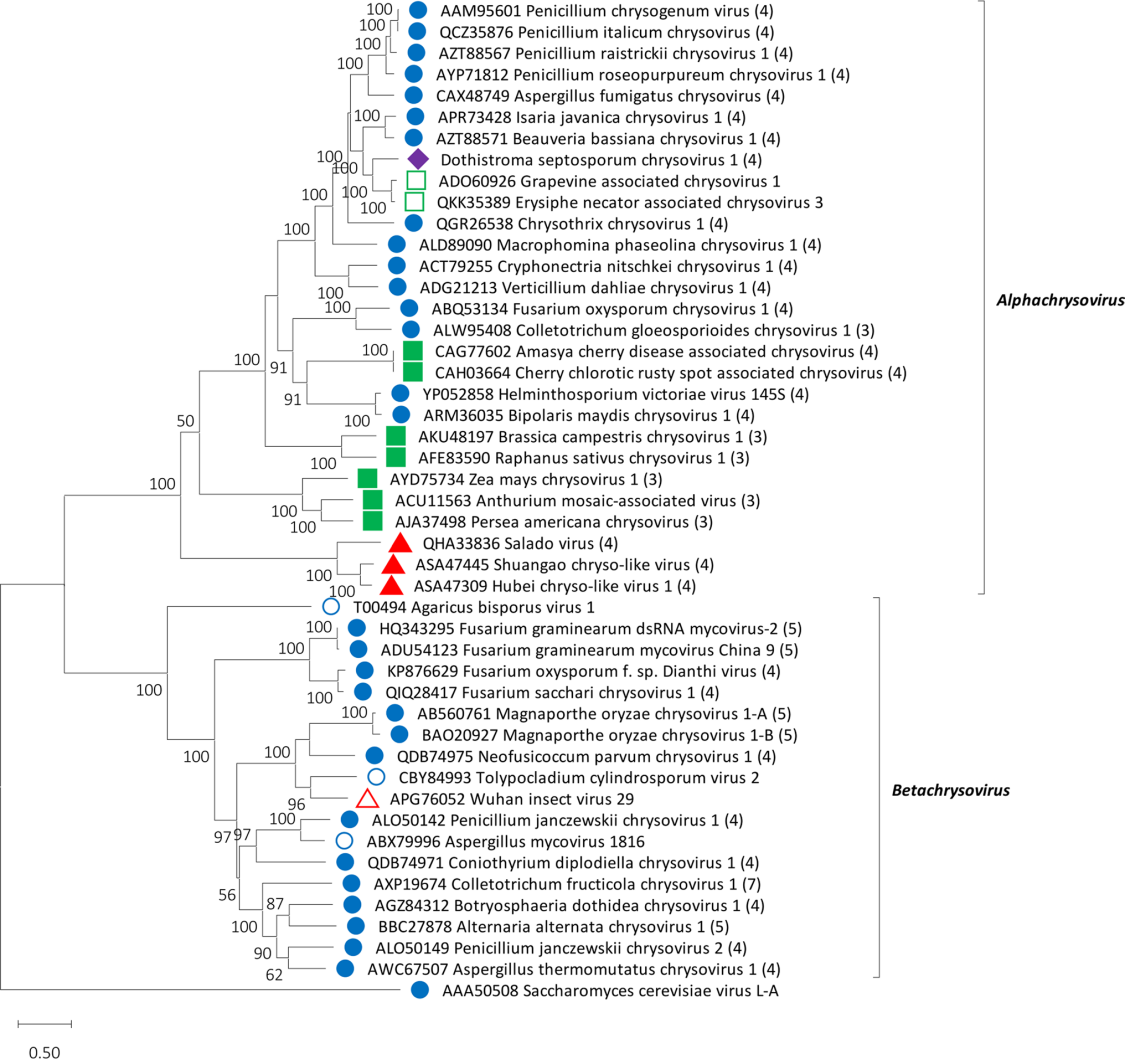
Phylogenetic analysis of the RdRP aa sequences of DsCV-1 and selected members of the family *Chrysoviridae*. A member of the family *Totiviridae* was used as an outgroup, and only bootstrap values >50 are shown. The number of dsRNA genomic segments is indicated, if known. Virus hosts or associations are indicated by circles (fungi), squares (plants), and triangles (insects); unclassified viruses are indicated by open shapes. The rhombus indicates DsCV-1.

In conclusion, DsCV-1 is a new member of the genus *Alphachrysovirus* in the family *Chrysoviridae* and is one of the three chrysoviruses reported whose alphachryso-P3-encoding dsRNA potentially produces an additional protein of unknown function. This is the first identification of a mycovirus infecting *D. septosporum*.

## Supplementary Information

Below is the link to the electronic supplementary material.

Supplementary file1 (DOCX 22 KB)

Supplementary file2 (FAS 66 KB)

## Data Availability

The sequences generated and analysed during the current study are available in the European Nucleotide Archive, project accession number PRJEB57200.
