# Supplementary material for: Identification and sequence determination of a new chrysovirus infecting the phytopathogenic fungus Dothistroma septosporum
Source: Arch Virol. 2023 Apr 18;168(5):144. doi: 10.1007/s00705-023-05768-9 (PMC10113357; doi:10.1007/s00705-023-05768-9)
Supplement: Supplementary file 1 — Supplementary file1 (DOCX 22 KB) [file 705_2023_5768_MOESM1_ESM.docx]

>RNA 1

TGATAAAAAACAAATAATCCTGCCACCTGAGGAGAATCGAAATCCCTGTGGGATAGTGGCAACACGGATTATTAACAAGAGACAAGTCACTAAGAACAAAACACACAACACAGACAAATTACACATAACACTCAACACAAACAATCTCAATTAACAAATTCAAATTACAGCAAATTTAAAACAATATGTCTGCCAGCCGTCAACCCCCCCGTTTGTCACAGCAAAAGAGGAAGGCATTGATCTCTCTGTCCCGGTTCAACCCACAGGAATCAAAGTTCCTAGGGAAAAATAAATCTGGTCAGAGCTATTCACAGCAGATTGAGCACTCAATGGCTAGGTATATTGGCATCAGAGAAAGAAGGGGTAACCTTTTTGCTTTCATACTACCGGCTGGTCACGGAAAAACAACAATTGCCAGGAAGTACGGATTCGTCGATGTAGATATGTTAATCAGCAGTCGTGAGCATGAGTACTTTGTCGAAAAGCGGCTGGCCATATTAGCAGGGGCAGATACATGGTCAGGTCACAACACCGAATGGTATGCTAGGTTGAATAGGACACTCGATCTGCTTGACTATTCGGTACCGGTGATAATACTGGTTCACACGGAGGAAACGGCACTGGAGCTGGGGGCTATGCCTATCGGTCGGTTTGTGCTGGAAAGATCTGTCTTTGAAAGAAATATCGCGGCAAGGGACCACCAAAGCAAGGTATTCTCAAGAATGAATTATGATACGTTGGTTACAAGTGCTTCAGTTCCAAATCAGGTGAAATGTAAGGACATGGATGAGTTAGAGGAGTGTATTCTAGCGGTCATGAATCTATCTCACATGCCTGTCGCATGCCCGCACAAGTTCAGCCGCGCCAACTGGAATGACACTTATCACAGGACCGTACCAGGCTGGGTTCTAACAGGTGAGAGAGCAGGCGATCGGTCGGTCGACATCAATTATCTACGGGAACTGTTTGACAAGGGACATGTGCCCAAGGAATGTGTAGACTACCACGTCAAGCATAGTTACGTACCAACTCAGTTTGACTTCGGTGTAACGATGTACGAGTGGTCAGCACTGCTAGCGACCTTCCCTCCAATTATGAACAAGCCCAGCCATTTCAACCCATTGGCTGACATGGTGGAGGTGTTCCCGCCTCGTGGCTCTAAAGAGTTGGTGAGAGCTAATGTTACCATGAGGCACCTTGTTCAGACATTTGACATCTTCTCACACGCTGACGCTTATGATATTGCTTCCCATCATGTCGGATCACCGCATGTATTTGTTACGTCAATACTGGCTCATTGGAAGGGTGTTCTTCAGGACACGCAAGTCGCGGACTTGATATTGCCCCTCTATAAGGTGTCTTTCCATGGTTGGGCAGATGCTATGAAGAGCCTACACTCATACACGCGAACAAGTCGGTTCTTCATGAACACTGAGATTACAGAGGAAGAACGCCAGAAGATAATGTACATGGATCTGCTAGTTGGAAGGTCGGAGTACAAAATAGATGAAATGGCCGAGGTAACACTTAGGTCATCAGACACCTACGACGCAAAACATCTATCCTTTGATCCTGACAGGCGCATTTACACTAACGAAATGTACAAGAAAGATTTCTCACTTAGTATGCAAGAAGCATACATGCGGATCAAAACCAATCCAAAACCAATTTTCCAACAGAACTTCAAAGATTTCTATAGTAGGCGGTCGACCTGGGTGACGAAAGGTGGCCTGGTGCAAAACACGCTCCCAAAGGAAATGAAGAAGTTTATGGCAACAATCTTGGATAGTGTGAGTAACACAATTGACGAAATAGAAGGCCGACACAACAAAAAATCTCTTTTTGAAGTCAGTGAGCTATTTGAAATACTGGTCGGGGTGAACAAAGAAAATTTCAATTTAACAAAAACGATGATAAAATACGAAGTGGGAGAGAAGTATCGAACGCTGCTTCCCGGTAGCCTGGCCCACTTCATTGTCTTTTCTTACATACTAATGCTTGCCGAGAAACAAGAACAAATAGGCAGTGTTCGCTTGAATGCTATGGGTGATGGGGACATACGCTACTTCGACCGAAAGATGTCAAGTGGCGTGTTCCACGTTCTTTATGACTGGGCTGACTTCAATGAGCAACATTCGGCTTGGGAAATGGCAGCTGTCATAAAGGAATTGGAAGTTGTTATGCCACATAATGCTGACTATCACCTCTACTGCGCCGCAATCGTTGAAGGCATGTTCTCTATGGGGCTGGAAGACCGGGATGGTCACCTTCACAAACTCTGGCGAGGGCTGTACTCAGGCTGGCGAGGTACTACCTGGATCAACACCGTTTTAAACTTTTGTTACGTTTCTATTGCCCTTAAGAATCTAGAACGGATCACGGGTCAGTCTGTTGTACTAATGGTTGATCACGGTGGTGATGATCTTGACTTGATGTTGAGCCAACCTAACGCAATGCCACAGTTCCTGGAAATTATGGACGATATGCTGTTCAAAGCAAACAAATGGAAGCAGATGTTTGGTTTGAGGTCTGAGTTCTTTAGGAACACAATATCAGGAGCCAGGGTATATGCAAGTCCAACGAGAGCATTAGCTAGTTTCGTGGCAGGCGATTGGGAAGGGGCTGGAGACGCAACTGTTAAAGAAAGAGTTGTCAGTTTGCTTGATCAAATAGGTAAGTTGAAAAGACGGGGTGTCAGCAATGATATGTGTCAGGGTTTTACCATGTGTTGTATATCACACTGGTGTAAAGTAAAAGAAGGTGAGGAATGGGTGAATTTACCACCATCCATACTTCATGGGAAGGTCGAACAAAATGGTCTGGGTGTGCCTGACACATACAACAAAGTCTGGGTGCTTAATAAGCCTGTGCCAGAGATAAGTGATGAATGGTACAAGTTAGTTGTCCCGGACTACAAAGCGAGCCGAGATTACGTAAAGGTACTGTCCCAGGAAGTTGAGCGTTTTACAATGGTGATTGAAAGGCAGGAAGACCTGGCGCGCAAATTATCAGAGGATTCGTACAATATAGAAAAGCGCGTAGACAGGATTGCGTGGCGTACGCTGCTGGATTTTGATGGGTACCCAATTGCATATGAAGATGCTATTCAGCCAATGGAGTCGGAGGAGGTGTTTGAGGCATTTCTCACATTCCCAGAAAATCCTGATAATGAGAGGAAGTTTGCCAAAGCAGCAAGGTATCAGGAGTATGTACAGTACCTGATGGTAGGTGACCGTCATATAACGAAAGAGGAATTGGTCGAAATCATGTCTGACGGGGAAGTGAGTTTAGCGGCTATAGAGTTCCAGGGAGATATCTACTACCAAAGACTTGTGCCTGAATTCATAAGCACTAGAGCACTTCTATTCTGTAGGATAGGAATTAATTTGGGTGAGTTCACAACAGAAGATGCTGACCACACGTTTAAGACAATATGTTGGATGGCGAGCAAGATATTTCGTCATATGATGTAAATATCAAACAATGAGTTATTTTTAACAACTATCCCTGAGGCTTAAAGCCGAACGGGAGAACTCACACACCCACAAGGGTGTGTGACAACCGAGTTTTAAAAATAAAGTGT

>RNA 2

TGATAAAAAACAAATAATCCAGATGCCAACTGAGATTATACACACATAGATCACACATTCAATAGATAGGCAATTTAATACTCACATTTCAAGTACAAAATGCCAACCTACAGTGAGGGGCATGTCATGGCACAGCTCATGGCCAAGTGGAGCATACAAAACGTAGGCTTGGTCAGTGATCAAGAAGTGAAGAAAGAAAATTGGCTTCATGATGGGAAGGTTTTGCCCGGGCCTAGATTGTTAGCTATGATCATGGATTACAAGAGGAAGGCCAAAATCTGTGCTGGTGTGGGTTATTCATATTCCACAGACGCATTTGGCCAATTAGACCAACAGTTTGTGAATCGTTTTTCGGATGAATTTTGGCAAATGAGTCAGCTAACTTATGGTATGTCTAAGATTGCTAGGGACCACGTCTCCACGATCGTATCGGACTTCAACGCATTGGGAGTGGCTGCAGCAACGGATATAAATGACATGCTTGATATGTTAGATGCTGTTGAAACATGGTCAAATAATAAGTAGATAACAACCGCCATGCCCCTGAGGAGATACGAAACCCCTGGTGGGTAGGGCAGGATAATAATACAGACCATACTCAAAACACACAAATCACACAATGGAACAGACAATTATATCATGGGTTCGTGGCGGCGGAGGTCGATCTGGGAAAGATAGGGATTCAAAGAAGAACCTAGATGTGAGTAAGAAGCAAGTATCATCAAGACGCGATGGAATTATAAGACCACCCAAGCCTAAGTTCGAAGGTGGGAAAACTAGGCGAAAAGAAACCAAAGAAAATGCTTCAGTGCGACGGTTCACAATGGGATCAAGTGAGATGCTACCTGCACCAACTACTGACCTGAGGAATTTTATAGGGGTAGTTGTACCACAGGGTCATGGAAAAACGATGCTGGCCAGGGAGGAAGGCTGGATCGATTTCGACTCATTGATATCTTCTCGATCGCTTGATTCACTGCGCGAAACAGCATATGATGAGATTAAAGCAGGCAGAAGTATCGAGGACGCGTCAACCAATTTCGCGGCTGAGGCACGCGAAACACTCAAGCTCCTCAACCCTGACTATCCAACCATATTGATGTGCCAAACATTCTCGTTGCTTGAGGCCATAGGCGTGGATTGCATTGGCGCAGTTGCAGTCAGACCAGACGTAGTGTTGAAACATAACAAGCGTAGGCAGCTACACGAGCTGTTGATGATAGAAAAAAACATTGAAGAGGTTGTAAACTATGATAGATGGGATAGTGCTGAAGTAGCCCTCATGGAAGGCATGGATGATGTACGCTGGTACATATATAATATATGTCAACACTTGGGCATACCTATTTCGCAGCCACACCTTTACTCAATGGTTGATGAGAATATACGGTCATGCAAGTACAAGACAGGCAAATACCCACCGCTAACCGAGGTCGTTTCGGACTACTATGCGGGTCTAATACCGAGGGAAGTGGTTGATCATCATGTTCAGGCTGCAGGGCTGAGGTCCTACCAGGGTTTCGGGTTTACCATGAATGACTGGGCAAAGGTTGTGGGCCACGCAAACTCTACACGAGGATCAACCACCTTCAGTGACATTGACTGGAATGCCTGGCCAATGTCATTGAAAGGTCTTTCTGAATCACTTGATCTAGCTCAGCATGACGACATCAGATTCATAATTGAAGCGCATAAGGGCGAGCATGAGAGGTTCATATTAGGCTTAATATTACATTGGAAAATGTTAGGATTAACATCTGGGCTGAAATCAAAACTTCTGCCGCTATATGCAGTCAAAAGAGTACACTGGGTTGCAGTCTTCTCAAAGGTCCGAGACGGGGTTCTTGCAAGCAACGGATTATTCGGTCAGCCGCTTACCGTTGAAGAACGCGAGCTCGTGATAAGTATGCGACTACTAGCTGCAGGGGAATTCTCACAATTGCAAAGGCTGCTTACCTCGGAACAAGGGTCATGCCCAAGGAGATCTCCGACACGGTTGATTCAAGACTCCGCTTCGAGAGGTCTCGATTCAGTGGTTTTTTGTGCTGACTCACATCAGGAGCGGGAGTTAGCTTTCAGCGAAATGCTTAAACAATCAAGAATGTCATCGCTTAATGAAGTTGACTGGGACGGAAAACTGACTAGACTGCAATCCATAGCCAAGTCCATAGGAAAGGATCTGAGCCTGCGTTGGCGTGATGAACAAAGGGGGTTGGAACGGGTTAGCCGCATACTGGCTGGCTTGCTCAAAAGATGGCATAAAGCGTGTTTAATAAGAGATGAGTGGTCTGATATGACGAATGCGCTACTAGAAGAATGCTCGGAACATGAAGCATTGGGGGACGCCATAGCAGCTATGCTGTCATGTAGCATACAGGAAGGTTCAGAAGGGCATGACTGGAGTGTTAGAGTAATGGAGGCACTGAAAGGTTTTGTCGTTTGTGGCCTGGTTTGCGAAGGTAAAGGAAAAGTAGTTATGCAACAAACGCATAAACTGCTGAGGCCCTGCGTCCTTGGTATGAACGAGGCGGAAATATGGTCCAAGGCTATTAGCCTAAATATACCTAGAGGTGCCCTAGGCTGCTTCTCGTCAGGCATAAGTCACTTGCAACTGTTGAATGAATTATGCGGGTGGTCGACTAATAAAACTGTCATGGTGATGGAAATGATAAACTCAAGCAGCTGGATGCCAAATATGTCAAAAAGAGCAACGTTGGCTGGACTATGCCGATGGAGGAATTTCTTCACTGATGTTCAGGAGAGATACATCTTCGAGAAAATAGCCGACAGTTATACAATAAGATCGATGGGTAGGTCATACTTGAAGGTTGAACACCGCCTAATGGAGCTGATATCAGTAAAAACTAGCAGTGGGGGCCTAGGTTGTGGTCCAACATTATACGACGCCAAAGTTAATAAAGGTACTGATGGTTTCTGGAACGGGCACGGAAAGATAGGGTTTGGAAGGAAGAGTAGGATGTCTGGCGATCCAGGGAGCATCGCAGATATGATCGAACTGTTTGAAAGGGATGATGGCCGTGAACAAAAAACAATAGCGCCCAAGAGTATATACGAAGTTGGGCTAGCATCACTACAAGTTATACGGGCAGACAATAAAAGTCATCTCAGCAGACATGCTAGACTACTAGTTAGGCTGGCTGAGGAACGGCAGAAGTAGTGATCAATAATGAGTTATTTTTAACAGCCGAAGGGTTACAAAACCTGAGGCGATCCTGCTTGTCTTCAAAGACAAGTAGCAACGGAGTTTAAAAATAAAGTG

>RNA 3

TGATAAAAAACAAATAATCCGTGCCACCTGAGGAGAATCGAAACCCCTGTGGGGTAGTGGCAACACACGGATTATAGACAACAACATTGAACAAAACAGATAAGATACCAAGACAAACAAACATAAATAACACAATCACAAACAATGTCTCAAATCAACTTCAGCAACGAACGTTATGATGAAAAAACGGCGGCGTTCAACGCATTGAGAAGTGGTGCCACAGCACTAAGGAGATCAAAGGCTCTAAGTGTCAAGTTGACGAGCTGGGATCCGGCGACAAGAGCAGGCGCTTCAAATTTCAGAGAGAAGCAGAATATGCTGGGGCGCGATATTGGAAGTGTCGCCAATTACTTTGATAACAAAAGAAGTTCGGCACTCGAGGTGATATGCAAGGATGAATTTACTGTCAACTACCAGATATACGGCGACATAAGAAGAGAGGCAGTATTTGGGCAGAATACCTTGTCCATTTTCTTCCCAATTAAATGGTCGCAATGCGAGGTTAACGTGAGTTTGTATCCTGATGTGTTGGACAAGCCTATACCCAGAGAGAAATTTACCACAGCTGCGAGAGAAGGTATACCTAATCGAGATGATATTGCCAAGGTGACGGGCTGGAACCGCAACGTCGTTCGGGACATTCAGGACACAGACATAAGCATGTTCAAAGTGCTGATAGGTCAGGTGATGGTTGGTCAGTCAAAGCTAACAAGGTTAGTCAAGGGGTTCCTTATGCTTTTGGAGTGCATGGAGAGGGATCACATAGATGTGGTACTCGATGTCCAAAATACGGTACTCTACAACCCCACACAAGTGTTGAACAGCTTCAGGGCCAATGGCCGTGCGTATGTTTACAACTCGAAGCCCTCATCTTCTGTTCACACAGCCGTGCTCTGGCGTATGTGCGGAGCGTACCCTCCACCTGAAATAAGAGGGTCTCACATTCAAATACCGGCCGACGGCGCCAACGTATTTATGGTGCTTGAGGGTGCAGTACCGGCCCAAGGTCAAAGAGTAAGGCTGACTCAGGGACTGATTTATGCAAGCATCATGGCGTATGCTATGGACGTTAGTTGCACTCAGCATCTACAGCAAGCACTGATTATTGCTTGTTCACTGCAGCAGAATCGATACTTTTCAAGGGTTCAGCTCCCTGCAGTAGTTTCGGTGATGGATTTGATGATTCCTGCATTTATGACGACTAGCTCAAGGTTAGACAAGCCAATACTGTCGATTGAGATGGCAACCTCGGTTGGAAGATTGCGTCAGATGCTCATGTTCATGAATGTAAAGGATGTCTTGACATCAGCAGAATTGAGCACATCACGAGGGTTTGATCCAGCACAAAGCATGAGATCATACATGAGCTCACAGGCTGCTCTGATCACACAAATGAGCAGCGAGATCTCAGCGCTATGTTTGGTAGAGGCCGCAATCAAGATGAAGGTTCACGAAGCCATGCGCGAGGATGATTTTAGTGATATACTAAACTTGTCGGCCTTTGAAGGCCTTTGGTTGTGTCAGGAAGGAACAAAGAGTGTTAAGAACGGGATCATATCAGCACTTGTTAATGGGGTATCTGACCTCAGTGGTGATATGTGCTCTTACGACATAGTACGCCGGGAGATGGATCTCGGTAGGATAGTCTATGACCCGGCGGCAATGCCCAAAGGAGCTTTCACTGTGGGATGGGTATGCGTATCACCATCGATCAAGAAAGCGGTACCAGCACCTCGAAAGAGATTGTCGAGACAGGTTACCCTCGTTCATCCTTGTGAGACAAAGCCGGGATCAACCTTACGGGTAAGCCGAAAGAAGCGATTTATGAGAAATGATCATTCTGGGGAGAATGAGACGTCTCGCTCACCAACCCCAGAGACAACGATACCTGTAAAACTTCGTTTTGGATCGCCTAAGCGCTCGAGATCAAGCACGAGGGATAGTGTACCACCGCCTTATGTGGAGGAAATATCGGAGACCAGCTCAACCGTATCATCCGAGGAAATATATAGCAGGACGCGGCTGGCCAACGAAGTAATGGAAAAGGTGAAGCGGAAGGAGTTCGTGCCACTGTCACCCATGACAGAAGTCACCTCGCCTACCACCACTCCTGTAACACCTACTCGCTCCGCAATAGACGCGATCGTGGGTAATACTGCGGCCCAGACAACGGATTATGAGAAATTAGAAGGCCTGGCCAAGGGCTCAAATGTAAAATTGAGTGGTAAAGAAGTAGGGTCGTTACTGGCAGTACTTAACAGGACGAAGGGGGCGGGCTTAATTGAACACGCGCGTGACATAACAACGCTTGTTGATACAATGGCTGAAAAACAAATGTTTCTGGACTATGACGGGCCTGCCATGTCGGGGAACTCCAATCTTGCTAAGAGAATAAATCAGTTGATCGATTTTGGGGCTAAGCGAGATACAACAGGCTCACAACTACGAAAGATGATGGAAGATAAAAAAGCGTGGAAGGGTGAAATCACAACTGCACCTATCGCAGGACTGAGGCATGTAGTCAATACACAGCCATGGATAGAGTTCTTAACGGCAGAAGGAATTGACATCAACGAGAAGATAGATGGGCGAATAATGTTACAAGTGTTAGCTGAAACTATGTCACGCTTAGAAGACACATTTGCACCTGGAGACACTAAACTAGTGCACGAATGGATCACTGGAACTTCAACGGTCAGATTTCCTATGGACATCACGAAGGAAGAGTTAACTACACTTGGAGCGCCATCAGTATTAAGTTACCGCGTCGCGCCATCCAGCTTTGGACCTCGTGAATTTACACAGGAGGATAGTAAGTATGTTGTCATGGCTGCCCGCATTAACAGATGCGTCTTTGATACTGACGTGATAGCTAACCTTTCGGGCAAATTCACCCTACCAAGTTGGATGATGGCTGGGCTAAGGGAGAACTACGGCTTGTTCCAGGGCAGGAAGAAGTAGGTTTGCAAAACCAACAATGAGTTATTTTAGAACTCTGGAGCCACAAGGCTACCAGGGGACCCCGTGAGCTTTTAAAGCGAGCGGCCTCGATGTTTTAAAATAAAGTGT

>RNA 4

T/CGATAAAAAACAAATAATCCTGGCCACCTGAGGAGAATCGAAACCCCTGCGGGGTAGTGGCAACACAAGGATTATAATACAACACACACAAATACCACGCCCACAACAAACACAAAAACACACATTACACAGAACAAATACACACGAATACACATAAGATGTCGGCTGTAGACAGAGTAGTTGACTACATGTCGGGGCTGCCAAGTGCAGCAGAACACGGTCACACGTATGCAACTGAATTCACAGATAGACTGCTTAAGGGTGACATAACCGTGATTGATGGTGTGGAGGAAGAGAAGAAAATGATCAGTAAAGGTGAAAAAGCAATCATGGCCAGGCTCAACTGCCCGGTGAAATTCACTAGGCAGGGTTTCTCACTCGATGCGAAGATCGGTGTTGGTGTCTCGGCCGAGCGACGGCAGTTTGTTAGGCGTGACAGGGTTGAAAACCTCAACTTTCAAGGTAGTCACGAGTGGTTTCTGGAACCAGCACGATCGAGGGCACTGTACTCTTCTAAAGAGTCGACGGTCCACTTGCTCGACGAGGCACAAAAACGGAAAATAATGGCCAAATGCCCAAATATGTCCGGTGCAGATAGCAGTGACGTGTATATGATGAGTGTTCATGAGCGTATATCAGTGCCAACTGACATGCGACATATAATGATGAAGATGTATCTCCTTCTCCAAGATTACAATCTTGCGGTTACTTCTAAATCCGAGTCTGTTGACATTGCACAGGATTTGGTATATCCAATGACTCAGAACGCTACACCTATGGACCGGTTAGCTGCAGTCACATTGCATTCAATTGTCATCGACGCAGAAGGGTTTACCAGTCAGGAATTGGGTCTGCTGGCGATAGCGGCTCAGGAATATCCGAGTGTATGGTATACACATGATAATATTTACACCAAGTGCCATATGGCAAAGGACGATCTTGTACTCGTATCAGACGGCGAAATACCGATAGACACTAGTATGCTATGGGGCTCACCAGATCGCTTGTATCAGATGATGTGGACTGTAGCGGCAAAGCTCAACTGTGTCAACCATCTAATATCCGCATTCGAATGCATGAGGGGTAAGTGTCAGATGATGGCAGATGTCTTCTCGAAAGTAGATGCCAATATAGTAAATTCAATGGTGCCGTTGAGTTACAGTATGGAGCATGCTTTCGGTGGCGCTGTGAGTAGAAACAGTGTGACAAATGCGCCTGGTTACTTCTCAAGTAGTATGGCACTAGTCTCTGACTTGCTCTACGGAATGACCTTCGAGGCCTCAGCCACATGTGTAGCCGAGTCGCTCGGTGCGTGCGGCAAGCTACTCTCGTCTAGCACGCCGTCTACCAATACGGTCATCAATGGCATAATGCGCGATTTTGGTCTGCAGCACACAAATTCTGAGGAAAACTACCTGCTGCAAAACTGGGACATTATGGCAGGGCGGCCAATAACCTGGGACTTTGGTCCGATATTGAAGGACTATGTGTTAGAATTATCATCAATGATAATCAATGGTACTGACATACAGGTACCACAACTGCTCCACGCAATACCAAGCTTGACGGCTGTGAATAGCTGTTATGGGCTGGCCCGCGGCTGGCGAGGACCCGCAGATTCAGTGCTGACTAGCAAGAAACAACGCGCTGACGACAGTGATGGTATGGCTAGCTTTGCGTGGATGATGGGTGAAAGGAAGGTACGACCACCGGTATTTTATAACAGGGTGGGTAAGAAACCTATTAATCTGAGTCATAATGAATACTTTCTACAGGCTGAGTGTGAGGGTAATCACGGAATAGGTGAAGTCCAATTCTGGCTGGGTGATTCAGTCGGCGGTAGGGTGGATGAAAACGAGGAAACAGCTTCGTCGCTTTACAGAACTGAGTATGCAGGCACCAAATGCTCGATAGTATTCAACAAATCCGAAGAGCGTTGGGTGACGCATTCAGTCAGAGACCCCCCGGGTCGTGAGACAATGGCGGTCGATAAAAACCCACCCGAAACACAGTCTCAGCCGATTAAGGAAGAGATACCTGAGAGAGCGTTGACCGGAGTAAATTGGGGTGCCGGTCGGAAATTGAACACAGAATCGGTATTCAAACACCTAGCCTCACTCTCGCGCAGCAATCAAATTGTACCCTCAAAAAGAGCCCGCCATATGCGTATGACGAGCAACGGGGGCGTGGCAGTGGCCCCGTACGTAATAGACGGAAATCAGGAGAGCCAAATGCTAACAAGAGGCAAGCAAGACTTAGCAGAGGGTGTTGAACTACAGTACGGTGTAATTGATGTGCCAGGTGATGGCAGATGCGGCATACACGCAATTGTAGAGGACTTGAAGGTTCATGGGTTAATACCGCCAGCCGATGTCGCCAAAACTACCGAATTCTTCTCGGGAGAAACAGCATCCAGCACGTTCCATGACGCTCAAGAGCTTGCAGCATTAGCACAAACTTGGGGAATGAACATGGATCTTATAGACGTGGGTACTAGGGCTGTACACAGATACGGAAATCAGGCAGACGCCCATACAATAACAATAGTTCGTGATGGGGCCCACTTTTCAGCAGCCAAGATCGGTGTCGGCTCTCAAAAAATGCCGATAGAGCATCTACATACACAAGAATGCACTCCCGAGGAATTTGTAGAAAAAGTCAAGAGCTACGGCAACCTGTTCGGTAAGCCTACCGAGTAATTGAGATTACTCAATGTGATCAAAGAATGAGTTATTTTTAGACTCTGCAGCCACAAGGCTGTTAGAGGAACCTGTGAACCTTTAAGGGGATCAGACCAGAGGTTTAAAAATAAAGTG
